# Psychosocial Factors Affecting Wellbeing and Sources of Support of Young Adult Cancer Survivors: A Scoping Review

**DOI:** 10.3390/nursrep14040293

**Published:** 2024-12-14

**Authors:** Erica R. Timko Olson, Anthony Olson, Megan Driscoll, Donna Z. Bliss

**Affiliations:** 1 School of Nursing, University of Minnesota, Minneapolis, MN 55455, USA; mbdriscoll23@gmail.com (M.D.); bliss@umn.edu (D.Z.B.); 2 College of Saint Benedict and Saint John’s University, Collegeville, MN 56321, USA; aolson004@csbsju.edu

**Keywords:** cancer, survivorship, psychosocial, wellbeing, young adult, nursing

## Abstract

Background/Objectives: To identify and analyze what is known about the psychosocial factors affecting the wellbeing and sources of support of young adult (YA) cancer survivors. Methods: The search strategy included Neoplasms, young adults, psycho* or emotional well* or mental health. The OVID Medline and CINAHL databases were searched. Included were cancer survivors (YA) ages 18–39 at the time of the study. The studies included qualitative and quantitative designs, written in English, and published between January 2016 and October 2024. The results were recorded according to PRISMA-ScR guidelines. Results: Thirteen studies with 4992 participants found psychosocial factors to be the most important influence on life satisfaction with social support the most decisive factor. This expands the results of previous reviews by including a variety of study designs and data collection tools to provide a comprehensive understanding of the YA experience. Psychosocial concerns affecting wellbeing led to social isolation, low connectedness with family and friends, and significant distress. Consistent with previous reviews, the greatest challenges to wellbeing were psychosocial needs, which included seeking and delivering information that is easy to understand but detailed, which can decrease frustration and anger, and needs to be readily available and accessible. Unlike older adult cancer survivors, YA survivors are more likely to have reduced psychosocial functioning compared to their peers and suffer from higher distress than their adult peers and non-YA cancer survivors with anxiety as the most reported symptom. Conclusions: Interventions need to be developed that lessen the impact of a cancer diagnosis and cancer treatments. The specific needs of YAs must be further researched and evaluated to determine specific interventions and the support needed during this crucial stage of cancer survivorship. Future research must also increase the focus on the racial and ethnic diversity of participants as well as prioritizing underserved populations and the impact of the COVID-19 pandemic.

## 1. Introduction

While cancer was once strictly a terminal disease, those afflicted are going on to live long lives following treatment. However, survivors often face a multitude of late effects of physical, psychological, social, and spiritual challenges that require specialized care. Young adults do not receive the follow-up care necessary to support their wellbeing and a specific focus on this population is required.

There are an estimated 17 million cancer survivors in the United States [1]. One in ten cancer survivors reported a decrease in their quality of life due to emotional problems such as anxiety, depression, fear of recurrence of cancer, and memory/concentration issues [2]. Both male and female cancer survivors, regardless of age at diagnosis, have an increased risk for neurological disorders, such as anxiety/neurotic disorders, eating disorders, personality disorders, psychotic disorders, and depression [3]. About 32% of cancer patients report needing additional psychosocial care to help with many of these challenges related to their survivorship [4].

The American Cancer Society estimates that 80,000 young adults (YAs) are diagnosed with cancer each year, about 5% of all cancers, and that 9000 will die. The Surveillance, Epidemiology, and End Results (SEER) Program reports that the 5-year survival rate for the YA population is 85% [5]. However, in the US, the survival rates for YAs have not seen the same level of improvement compared to children and older adult survival rates in the last several decades [6]. Psychological distress has been reported in approximately 83% of AYA cancer patients with younger, divorced, females at the highest risk for distress [7].

YAs already face unique challenges due to this transitional period of their life, from facing decisions about what to do after high school, living on their own, financial independence, obtaining employment, establishing careers, and peer relationships, changing roles in their families, and starting families. They also face the long-term physical, psychological, and social effects of survivorship [7]. Aside from these social and personal milestones, YAs also face a multitude of psychosocial needs such as the development of body image and self-esteem, shared decision-making, and managing risk-taking behaviors [8]. Challenges to psychosocial wellbeing can include pain, stress, anxiety, hopelessness, feeling down, and worries. Social needs are defined by love, family support, family relationships, going out with friends, and relationships. YAs’ coping is often associated with peer support, underlining the importance of social interaction for this group [9]. Unlike older adult cancer survivors, YA survivors are more likely to have decreased psychosocial functioning compared to their peers [10].

Cancer treatment not only requires remedies for physical ailments but psychosocial care as well. Psychosocial care is an approach focused on the influence of the social community and psychological factors that have an effect on wellbeing. Psychosocial interventions for YA cancer patients improve their overall mood, and social and sexual relationships, with these benefits lasting longer than the duration of the intervention [11]. Social support is key for this age group, and group interaction programs can be useful in improving self-esteem and decreasing powerlessness [9]. Kwak [12] found that the levels of distress among YA cancer patients are high at the time of diagnosis and during their transition to survivorship. Currently, there is evidence showing the need for and support for standardized, evidence-based, psychosocial treatment guidelines for cancer patients [13]. With more than eight out of ten YAs experiencing psychological distress, there is a significant need for greater psychological support throughout their treatment and beyond remission.

There have been several new studies reporting the psychosocial needs of YAs since a previous review of these needs was published in 2017 [14]. Other reviews, 2013–2016, focus on studies of interventions to support the physical, psychological, and social wellbeing of YA cancer survivors [15,16,17]. Cancer survivorship has changed significantly since the latest review with advances in early detection, highly effective immunotherapy treatments, and many more survivors. The current models of survivorship care do not meet the needs of this population. This scoping review addresses the gap in the literature regarding the current psychosocial needs of YAs; results can serve to direct and evaluate the focus of future interventions. The purpose of the scoping review was to identify and analyze what is known about the psychosocial wellbeing and sources of support of YA (18–39-year-old) cancer survivors.

## 2. Materials and Methods

### 2.1. Search Strategy

A literature search was conducted to identify studies reporting on the psychosocial factors affecting the wellbeing and sources of support for YA cancer survivors aged 18–39. This search strategy was devised in consultation with a medical research librarian and included iterations of the following terms: Neoplasms and young adults and psycho* or emotional well* or mental health. The databases searched included OVID Medline and CINAHL and were last searched in November 2024. The searches were limited to human subjects, the English language, and peer-reviewed articles available in full text. The PRISMA checklist with extension for Scoping Reviews (PRISMA-ScR) was used as a guide to writing this scoping review. A review protocol does not exist for this review, and it is not registered.

### 2.2. Inclusion Criteria

This review focused on studies of the population of cancer patients between the ages of 18 and 39 years (YAs) at the time of the study. This age range was selected because young adults have greater autonomy and independence from parents/guardians, which can have a substantial impact on theirs psychosocial needs and choices. Studies whose research questions, aims, and purpose were focused specifically on YAs’ psychosocial wellbeing, needs, and support were included. The study designs could be qualitative or quantitative and a comparison group was not required. The studies needed to be primary research, written in English and published between January 2016 and October 2024.

### 2.3. Exclusion Criteria

Intervention and pilot studies were excluded as they did not meet the intended purpose of the scoping review. Editorials, review articles, dissertations, non-peer-reviewed articles, and tests of questionnaires/tools were also excluded. 

### 2.4. Selection Criteria

**Figure 1:**
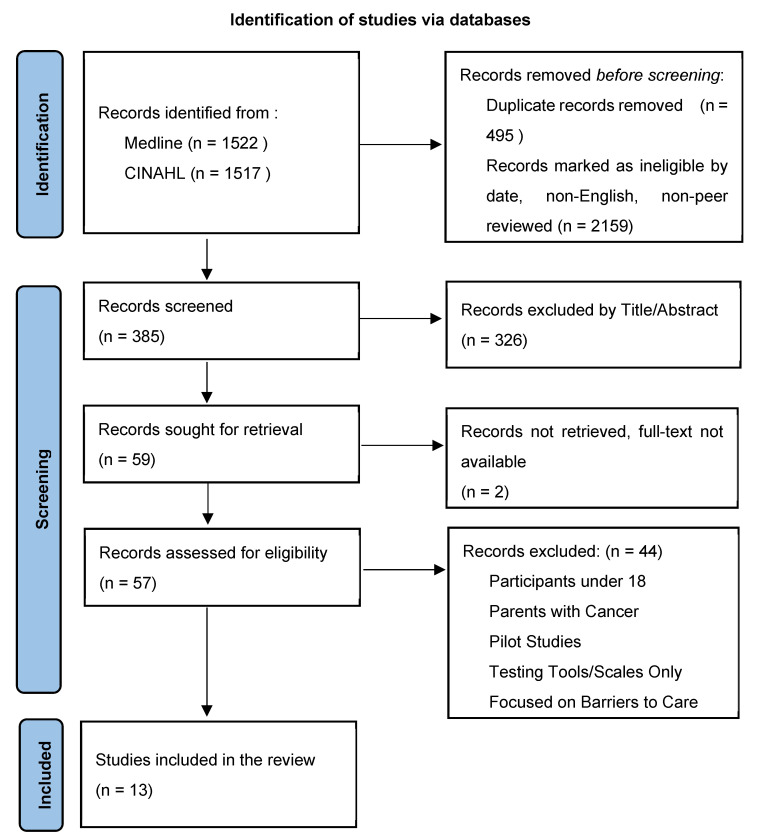
Flow diagram.

A total of 3039 records were identified through two database searches. After the removal of 2654 records that were duplicates, non-English, published before 2016 and after October 2024, and non-peer reviewed, 385 records were screened by two independent reviewers. The reviewers discussed discrepancies and came to a mutually agreed-upon decision. An additional 326 records were removed after the review of the title and/or abstract and 59 records were sought for retrieval. An additional two records were not available in full-text format, resulting in 57 full-text records reviewed. An additional 44 records were removed due to the age of the sample, focus on parents with cancer or barriers to care, pilot or intervention studies, and testing scales/tools. Thirteen studies were included in the review; the process is summarized in the flow diagram (Figure 1).

### 2.5. DataAbstraction

Data extraction was performed by two independent reviewers and included the author, year, research question, aims, or purpose; participants; intervention; instruments; and results. Data from each study were summarized and synthesized when possible (Table 1) based on similar characteristics and measurements.

## 3. Results

### 3.1. Study Characteristics

The total number of participants in the 13 studies reviewed was 4992 with an additional 6050 cases matched with older adults. The study with the smallest sample had 18 participants and the largest sample had 2045 participants. Two studies contained results of the same sample. The ages of the participants ranged from 18 to 39 years. Most of the studies were conducted in Germany (*n* = 7) with the remainder in Canada (*n* = 1), the United States (*n* = 3), Norway (*n* = 1), and South Korea (*n* = 1). The number of male participants was 1823 (36.5%), with one transgender man, and the number of female participants was 3168 (63.5%). Eleven studies included both males and females. There was one study with only males [30] and one study with only females [23]. Only three of the studies, all from the United States, reported race, with over 80% of the participants being white and minimal representation from African Americans, Hispanics, Asians, and other races reported. In all 13 studies, the diagnosis of cancer was during childhood, adolescence, or young adulthood. The three most common types of cancer in six of the studies were breast, leukemia/lymphoma, and gynecological cancers. Two studies addressed leukemia, lymphoma, and/or multiple myeloma [26,27]. Breuer [18] included YAs with breast, testicular, and lymphoma. Soleimani [30] included only testicular cancer survivors. Iannarino [22] examined primarily leukemia, testicular, and head/neck cancer survivors. Buro included most cancer types [19] while Li et al. included blood, brain/spine, and breast [25].

### 3.2. Study Designs

Regarding the designs of the studies, three were secondary data analysis (23.1%), seven were observational surveys (58.8%), and three studies had qualitative designs (23.1%).

### 3.3. Aims

The aims of the 13 studies included describing social support and distress/stress identification, comparing mental health predictors to health behaviors, or comparing the characteristics of YAs with non-YA participants. Six of the studies asked for sources of social support, the experiences and satisfaction of that support, and how they helped or hindered their experience. The results were used to develop recommendations for additional care and support [18,19,21,22,25,28]. Three studies looked specifically at distress/stress [20,29,30]. The authors measured anxiety, mental health disorders, and psychological distress, and how these compared with the physical/physiological function of the body. One study examined the association between mental health and health behaviors [19]. The final three studies compared the psychosocial aspects of care of YAs with cancer to non-YAs (elderly and older adults) with cancer [26,27,29].

### 3.4. Data Collection

A general assessment was conducted in the studies using several different assessment tools. The Canadian Problems Checklist (CPC) was used in two studies [29,30] and has not been validated. The following were each used in just one of the remaining studies: the Functional Assessment of Cancer Therapy, Bone Marrow Transplantation (FACT-BMT) [26], the Composite International Diagnostic Interview for Oncology (CIDI-O) [20], the Life Satisfaction Questionnaire (FLZ-M) [24], the Perceived Adjustment to Chronic Illness Scale (PACIS) [24], the Human Activity Profile (HAP) [26], and Short Form (SF-36) [26]. The scales have been tested extensively and determined to be valid and reliable.

Psychological/social screenings were performed in each of the studies. Two studies used the Hospital Anxiety and Depression Scale (HADS) [26,28]. Depression and anxiety were measured using the depression screener (PHQ-9) [20] and anxiety screener (GAD-7) [20], respectively. Stress and distress were measured using the Perceived Stress Scale (PSS-10) [19,23], PROMIS Anxiety and Depression [19], Adjective Measure (24-AM) [26], the Life Orientation Test—Revised (LOT-R), and the Distress Thermometer [28]. Psychosocial and social support were measured using the Illness Specific Social Support Scale (ISSS-8) [19] and Berlin Social Support Scales (BSSSs) [26], and two studies used the PsychoSocial Screen for Cancer-Revised (PSSCAN-R) [29,30]. One study measured the stress response by cortisol in saliva samples [23]. Finally, one study used the Eating Beliefs Questionnaire (EBQ-18), the Dietary Screen Questionnaire (DSQ), and the Godin–Shepard Leisure-Time Physical Activity Questionnaire (GSLTPAQ) [19]. These scales are used for psychological and social screenings, have been tested for validity and reliability, and were found to be adequate.

There were also researcher-developed questionnaires in both quantitative and qualitative studies that included demographics, general information [27], job-related situations [18], social support [18,21,22,25], psychosocial aspects [28], psychological counseling [28], social-legal counseling [28], and other medical psychosocial care [27].

### 3.5. Findings

#### 3.5.1. Psychosocial Support

Psychosocial factors were the most important influence on life satisfaction with social support being the most decisive factor [24]. The YAs found many factors that were a positive support. They felt supported by two or more people [24], with partners and close family members as the main support [21,22]. Emotional support was also reported as very important and shown by presence, phone calls, encouragement, confidence in recovery, listening, distraction, and being treated normally [18,22]. Unconditional support and empathy were also valued [18,21]. Informational support was important, and the needs included wanting more information on their body shape/sexuality, coping strategies, relaxation techniques, and their relationships with family and friends [27]. Negative sources of support also included family and friends [18]. Friends often distanced themselves and had difficulty coping with the situation [18] and the YAs reported negative feelings related to pity, sympathy, rudeness, and excessive monitoring [22]. Social connectedness was found to be a mediator between social isolation and depressive symptoms [19]. Many participants reported limited communication with other YAs and some preferred conversation with older or younger cancer survivors rather than people their age [18]. They preferred to have one-on-one conversations in quiet environments at a café or on the Internet rather than in a group [18,26]. These conversations can have both positive and negative consequences for cancer survivors [18,19,21].

#### 3.5.2. Psychological Factors Affecting Wellbeing

Many psychological factors were measured, as they can have a significant impact on wellbeing. About half of the YAs reported psychological issues [20,27,28]. They reported severe stress [19,23], depression, and anxiety [20,25,29,30] with women and the older YAs having higher distress [20]. The YAs were bothered more about their treatment success, future, and emotional role functioning than the elderly patients [26]. Perceived stress, anxiety, and depression were also associated with increased sugar intake and negative eating beliefs, while perceived stress and depression were associated with decreased vegetable intake [19].

#### 3.5.3. Psychosocial Concerns Affecting Wellbeing

The primary psychosocial concerns of the YA cancer survivors were fear/worry, understanding their illness, frustration/anger, sleep, sadness, finances, and work/school [29,30]. The YA survivors reported social isolation with low social connectedness as a significant contributor to social wellbeing [25]. They had higher rates of distress than the non-YA cancer survivors and adult peers with more fears/worries, concerns about work/school and finances, anger, and a lack of understanding of the disease [29,30].

## 4. Discussion

This scoping review described the most current findings about the psychosocial wellbeing needs and sources of support for YA (18–39-year-old) cancer survivors across 13 studies. There have been significant advances in technology and cancer treatments within the last decade, with new treatments, such as immunotherapy, becoming more widely available to young adult cancer survivors. This supports reviews about the most recent results about the wellbeing and support of YAs. This review’s focus on YAs aged 18–39 years, who have unique needs based on the critical developmental milestones that occur in this age group, refine what is published in the literature, as previous reviews also included adolescents aged 15–17 years [31]. We classified the reviews based on three key areas: psychosocial support, psychological factors affecting wellbeing, and psychosocial factors affecting wellbeing. Due to the significant milestones and developmental needs of young adults, these survivors experience different challenges than younger or older cancer survivors. Young adults are also underrepresented in research studies.

The studies used three different study designs, secondary data analysis, observational surveys, and a qualitative design to provide a holistic understanding of the literature that improved the applicability of the review. Findings show that psychosocial factors were the most important influence on life satisfaction following a cancer diagnosis. YAs need support from close family members and friends through regular in-person contact and phone calls. This is consistent with a past review showing that social support from peers and family, as well as connecting with other YA survivors, is the most important supportive care need [14,31,32,33]. Also, negative support from friends, family, and peers can lead to social isolation and an increase in social isolation as previously reported [31].

Psychological factors have a significant effect on the wellbeing of the young adult population. The results of this review expanded on previous findings about the negative impact of financial concerns, financial difficulties, and cost barriers that exist for this population [31,33] and disruption to education plans and limitations for employment due to health issues [31]. YA cancer survivors experience feelings of vulnerability, frustration, anger, and fear, as well as the need for more information about their diagnosis and treatment to address common gaps in understanding [14,33]. Unique findings of this review were the prevalence of psychological distress and its characteristics in YA cancer survivors [20,25,28,29,30]. YA cancer survivors suffer from higher distress than their adult peers [29] and non-YA cancer survivors [30] with anxiety have distress as their most reported symptom [20,29,30]. There is an immediate need for increased follow-up care support to address psychological outcomes. Mental health support, lifestyle changes, and coping strategies are important to address in addition to physical activity when planning interventions for this population; lifestyle interventions that address dietary needs in particular seem appropriate [19] to support psychological wellbeing.

Psychosocial concerns affecting wellbeing led to social isolation and low connectedness with family and friends, and caused significant distress [25,27,29,30]. Consistent with previous reviews, the greatest challenges to wellbeing were psychosocial needs that included the seeking and delivery of information to decrease fear/worry and increase the understanding of a cancer diagnosis. Information that is easy to understand but detailed can decrease frustration and anger and needs to be readily available and accessible [26,30,32].

### Strengths and Limitations

A strength of this review is that the evidence is from both qualitative and quantitative studies, providing richer and more comprehensive insights than would be available from studies using quantitative data collection tools alone. The limitations are the lack of availability of interview guides in the publications of the qualitative studies, and many of the studies used data collection tools developed by the researchers for their studies were not tested for validity and reliability. The racial and ethnic backgrounds of the samples were underreported and were only included in the three studies completed in the United States. This significantly reduced the generalizability of the findings. The lack of published articles that addressed COVID-19 as an influence on wellbeing outcomes suggests that the previous years have been challenging to conduct research within this vulnerable population. This was a scoping review intended to evaluate multiple study designs and an evaluation of the breadth of recent studies in the field and, consistent with scoping reviews, was not intended to do an in-depth quality appraisal of the individual studies but rather an analysis of the included studies.

## 5. Conclusions

This review expands the results of a review by Warner [31], as the latest studies used a variety of study designs and data collection tools to provide a more comprehensive understanding of the YA experience beyond the scope of the Health-Related Quality of Life and Symptoms questionnaire used in studies in previous reviews [31,33]. Results show that YAs continue to experience many of the same psychosocial factors that can have a significant negative impact on their wellbeing as reported in past studies [33,34] with greater psychosocial challenges and increased findings of psychological distress. Interventions need to be developed that lessen the impact of a cancer diagnosis and cancer treatments. Findings expand the understanding of how family support, peer support, and support by other cancer survivors can have a positive impact on psychosocial wellbeing [35]. The specific needs of young adults must be further researched and evaluated to determine the specific interventions and support needed during this crucial stage of cancer survivorship. These findings will guide the development of future interventions to address wellbeing unique to YA cancer survivors. These interventions must be designed specifically to promote the development of supportive family and peer support while addressing the barriers to wellness that include finances, education, employment opportunities, and diet. Future research must also increase focus on the racial and ethnic diversity of participants as well as prioritizing underserved populations and the impact of the COVID-19 pandemic.

## Figures and Tables

**Table 1:** Research summary.

Author, Year, Title	Study Design	Research Purpose	Participants	Method	Data Collection	Results	Types of Cancer
Breuer, N. et al. (2017) How do young adults with cancer perceive social support? (Germany) [18]	Qualitative Cross-Sectional Study with Semi-structured Interviews	Which source of social support for YA? What is helpful and non-helpful social support for YA? How do YAs experience exchanges with other YAs, and what ideas do they have regarding these interactions?	*n* = 18	Interview	Interview guide of 10 narrative questions and prompts.	Helpful support—all felt supported by at least 2 or more people; emotional support—presence and phone calls, instrumental and informational support; negative support—sources (friends/family Distance), friends; inadequate behavior—difficulties dealing with the situation; limited participation interchange with other YAs (preferred older or younger, not the same age).	1. Breast 2. Testicular 3. Lymphoma
Buro, A. W., Stern, M. and Carson, T. L. (2023) Reported Mental Health, Diet, and Physical Activity in Young Adult Cancer Survivors (United States) [19]	Cross-Sectional study	What is the association between reported mental health, eating beliefs, and health behaviors in YA cancer survivors?	*n* = 225 Race/Ethnicity White = 78% Black or African American = 11.2% Non-Hispanic = 84%	Survey	Demographics, Perceived Stress Scale (PSS-10), PROMIS Anxiety and Depression, Eating Beliefs Questionnaire (EBQ-18), Dietary Screen Questionnaire (DSQ), Godin–Shepard Leisure-Time Physical Activity Questionnaire (GSLTPAQ)	Perceived stress, anxiety, and depression were associated with increased sugar intake and negative eating beliefs. Perceived stress and depression were associated with decreased vegetable intake. None of the mental health predictors were associated with physical activity after adjusting for covariates.	1. Leukemia/Lymphoma 2. Breast 3. Thyroid
Geue, K. et al. (2018) Prevalence of mental disorders and psychosocial distress in German adolescent and young adult cancer patients (Germany) [20]	Cross-Sectional study	This study provides the prevalence data of mental disorders (4-week, 1-year, lifetime) and psychological distress in adolescents and young adults (AYAs) with cancer.	*n* = 302	Survey	Depression screener (PHQ-9) Composite International Diagnostic Interview for Oncology (CIDI-O). Anxiety screener (GAD-7).	A total of 29.5% of AYAs had increased depression symptoms; 20.8% had increased anxiety; women and older AYA had higher distress; mental disorders of any kind—46.7%; anxiety—24.4%; adjustment disorders—14.1%. The lowest rates were alcohol dependence and somatoform disorders.	1. Breast 2. Leukemia/ Lymphoma 3. Gynecologic
Hauken, M. A., and Larsen, R. M. B. (2019) Young adult cancer patients’ experiences of private social network support during cancer treatment (Norway) [21]	Qualitative	To explore young adult cancer patients’ experiences of support from their private social network during cancer treatment.	*n* = 20	Interview	Qualitative interview guide	Partners and close family were the main social support; most lacked peer support. Unconditional support and empathy were the most helpful. Mapping social networks is important for nurses to support cancer patients.	1. Breast 2. Leukemia/ Lymphoma 3. Gynecologic
Iannarino, N. T., Scott, A. M., and Shaunfield, S. L. (2017) Normative Social Support in Young Adult Cancer Survivors (United States) [22]	Qualitative	How did the normative perceptions of social support function to hinder and assist in coping with the cancer experience?	*n* = 30 Race: White = 83.3% Mixed Race = 0.67% African American = 0.03% Hispanic = 0.03% Asian = 0.03%	Interview	Qualitative interview guide	Effective support from peers/loved ones—treated normally; ineffective support—pity, sympathy, rude, excessive monitoring.	1. Leukemia/Lymphoma 2. Testicular 3. Head/Neck
Kim, J. et al. (2020) Psychosocial stress and ovarian function in adolescent and young adult cancer survivors (South Korea) [23]	Cross-Sectional study	It was hypothesized that there would be a negative association between psychological stress and ovarian function in AYA survivors. What is the relationship between self-reported psychosocial stress and the diurnal saliva cortisol levels with gonadotropins, ovarian steroids, and menstrual patterns?	*n* = 377	Survey and salivary samples	PSS-10, cancer, and reproductive characteristics; DBS, saliva, FSH, and LH measured by DBS ELISA; cortisol measured by ELISA; estradiol measured by liquid chromatography–mass spectrometry	PSS-10 showed severe stress in 5.3% of the participants, cortisol was negatively correlated with the PSS-10 scores, and the PSS-10 and Cortisol were not associated with FSH, LH, estradiol levels, or menstrual pattern. No cortisol measures were related to ovarian function measures.	1. Breast 2. Leukemia/ Lymphoma 3. Gynecologic
Leuteritz, K. et al. (2018) Life situation and psychosocial care of adolescent and young adult cancer patients—study protocol of a 12-month prospective longitudinal study (Germany) [24]	prospective longitudinal study	Determine the psychosocial life and supportive care situation of AYA cancer patients, describe risk groups, and develop recommendations for their psycho-oncological care and support.	*n* = 524	Survey	The Life Satisfaction Questionnaire (FLZ-M), Perceived Adjustment to Chronic Illness Scale (PACIS), Illness Specific Social Support Scale short version-8 (ISSS-8)	Lowest LS: financial and professional situation, family planning, sexuality, and the LS significance at baseline were the sociodemographic, medical, and psychosocial variables. At follow-up—psychosocial factors remained most important for LS; social support was the most decisive factor associated with LS at both points in time.	1. Breast 2. Leukemia/ Lymphoma 3. Gynecologic
Li, X. et al. (2024) Social Isolation, depression, and anxiety among young adult cancer survivors: The mediating role of social connectedness (United States) [25]	Cohort Study	Examine social the wellbeing in relation to psychological distress in a large cohort of YA cancer survivors.	*n* = 304 Race/Ethnicity Non-Hispanic Black = 7.2% Non-Hispanic White = 68.4% Hispanic = 19.1% Other = 5.3%	Survey	Demographics, clinical characteristics, PROMIS: social isolation, social connectedness, psychological distress	More social isolation was related to less connectedness, more depressive symptoms, and more symptoms of anxiety.	1. Blood 2. Brain/Spine 3. Breast
Pulewka, K. et al. (2017) Physical and psychological aspects of adolescent and young adults after allogeneic hematopoietic stem-cell transplantation: results from a prospective multicenter trial (Germany) [26]	Secondary data analysis	Analyze physical functioning, psychosocial aspects, and quality of life in AYAs in comparison to elderly patients undergoing alloHSCT	*n* = 271	Survey	Functional Assessment of Cancer Therapy-Bone Marrow Transplantation (FACT-BMT) Human Activity Profile (HAP) Short Form-36 (SF-36), 24-Item Adjective Measure (24-AM), Life Orientation Test—Revised (LOT-R) the Berlin Social-Support Scales (BSSS) Hospital Anxiety and Depression Scale (HADS)	Psychosocial—similar scores for anxiety and depression, equal values for emotional wellbeing, functional well-being, and social and family well-being. They were optimistic and less conscientious; the AYAs were bothered more often about the treatment’s success and future, and had higher emotional role functioning. They maintained their physical functional abilities but experienced the same comorbidities; AYAs cared more about their future, treatment concepts, and emotional role functioning than the elderly patients.	Leukemia/Lymphoma
Pulewka, K. et al. (2020) Clinical, social, and Psycho-oncological needs of adolescents and young adults versus older patients following hematopoietic stem cell transplantation (Germany) [27]	Cross-Sectional study	Examining the unmet informational needs among those with HSCT and evaluating whether these needs differ between AYA and non-AYA patients.	*n* = 30	Survey	The questionnaire developed by staff 4 categories: demographics, general informational requirements, need for advice on medical psychosocial aspects, the preferred channel of information	Unmet needs: 62.5% medical, 41.1% psychological, and 64.9% social care issues. Over 1/2 AYAs reported psychological issues—psychosocial needs (body shape/sexuality, coping strategies, relaxation techniques, family relationships, and friends, preferred one-to-one conversations in a quiet environment, little interest in groups).	1. AML 2. MM 3. ALL
Sender, A. et al. (2021) Psychosocial aftercare of adolescent and young adult cancer survivors in Germany: Awareness, utilization, satisfaction and associated factors (Germany) [28]	Cross-Sectional study	Assess awareness of and utilization of and satisfaction with psychosocial care of AYAs in aftercare	*n* = 514	Survey	Self-developed questionnaire on psychological counseling (PC) use, social–legal counseling (SLC), and other psychosocial care (OPC), the Distress Thermometer, Hospital Anxiety Depression Scale	Females and those with high anxiety utilized services.	1. Breast 2. Leukemia/ Lymphoma 3. Gynecologic
Smrke, A. et al. (2020) Distinct Features of Psychosocial Distress of Adolescents and Young Adults with Cancer Compared to Adults at Diagnosis: Patient-Reported Domains of Concern (Canada) [29]	Cross-Sectional study	Psychosocial needs of AYAs compared to older adults with cancer at diagnosis	*n* = 2045 case matched with 6050 older adults	Survey	Canadian Problems Checklist (CPC), PsychoSocial Screen for Cancer-Revised (PSSCAN-R)	Top 5 concerns: fear/worry, understanding illness, sleep, sadness, and finances. Reported higher symptoms of anxiety at baseline (moderate and severe).	1. Breast 2. Leukemia/ Lymphoma 3. Gynecologic
Soleimani, M. et al. (2021) Patient-reported psychosocial distress in adolescents and young adults with germ cell tumors (Germany) [30]	Cross-Sectional study	Hypothesized that AYAs experience more anxiety and distress in emotional practical and physical domains.	*n* = 349, AYAs *n* = 227	Survey	Health Assessment Form (HAF), PsychoSocial Screen for Cancer-Revised (PSSCAN-R), Canadian Problem Checklist (CPC)	AYA’s top 3 concerns: finances, work/school, frustration, and anger, clinical anxiety higher rate than control with more fears, worries, concerns about work/school, lack of understanding of disease, finances, frustration, and anger.	Testicular

## Public Involvement Statement

There is no public involvement in any aspect of this research.

## Guidelines and Standards Statement

This manuscript was drafted against the PRISMA-ScR guidelines for Scoping Reviews.
